# Efficacy of Tranexamic Acid in Reducing Blood Loss During Burn Surgery: A Systematic Review and Meta-Analysis of Randomized Controlled Trials

**DOI:** 10.7759/cureus.88353

**Published:** 2025-07-20

**Authors:** Mullapudi Lokesh, Shamsha Hirani

**Affiliations:** 1 Medicine and Surgery, Gomel State Medical University, Gomel, BLR; 2 Cardiology, Baqai Hospital, Karachi, PAK

**Keywords:** blood loss, burn surgery, hemostasis, meta-analysis, tranexamic acid

## Abstract

Burn injuries remain a major global health challenge, with surgical interventions often requiring blood transfusions due to substantial intraoperative bleeding. Tranexamic acid (TXA), an antifibrinolytic agent, has demonstrated efficacy in reducing perioperative blood loss across various surgical specialties, but evidence in burn surgery remains limited and inconsistent. This systematic review and meta-analysis aimed to evaluate the hemostatic efficacy of TXA in burn patients undergoing surgical procedures. A comprehensive literature search was conducted across PubMed, Embase, Web of Science, and Cochrane Central Register of Controlled Trials (CENTRAL) databases from the inception of databases to June 2025. Randomized controlled trials comparing TXA to placebo or no intervention in burn patients undergoing surgical procedures were included. Data extraction focused on intraoperative blood loss, hemoglobin changes, and hematocrit changes. Quality assessment was performed using the Cochrane Risk of Bias 2.0 tool, and meta-analyses were conducted using random-effects models. Four randomized controlled trials encompassing 264 participants were included, with 130 patients receiving TXA and 134 controls. The pooled analysis demonstrated that TXA significantly reduced intraoperative blood loss with a mean difference of -181.60 mL (95% CI: -267.34 to -95.86; p < 0.0001). TXA was also associated with significantly smaller declines in hemoglobin levels compared to controls (mean difference: -1.02 g/dL; 95% CI: -1.39 to -0.65; p < 0.00001). These findings suggest that TXA provides meaningful hemostatic benefits in burn surgery, although larger multicenter trials are needed to validate these results and assess long-term safety outcomes.

## Introduction and background

Burn injuries represent a significant global public health challenge, accounting for an estimated 180,000 deaths annually, with the vast majority occurring in low- and middle-income countries [1]. Survivors of severe burns often require multiple surgical interventions, including early excision and grafting, which are essential to reduce morbidity, infection, and mortality. However, these procedures are frequently associated with substantial intraoperative blood loss, typically ranging from 20% to 40% of total blood volume (TBV) or 0.5-1.5 mL per cm² of excised burn area, with blood loss patterns varying significantly according to the timing of surgical management, while delayed procedures may present different bleeding profiles due to tissue maturation and scar formation. This substantial blood loss necessitates blood transfusions that carry risks such as transfusion-related reactions, alloimmunization, and transmission of infectious agents [2-3]. Consequently, strategies to minimize surgical bleeding are critical in optimizing outcomes in burn patients.

Tranexamic acid (TXA), a synthetic derivative of the amino acid lysine, functions as an antifibrinolytic agent by reversibly blocking lysine-binding sites on plasminogen molecules, thereby inhibiting the degradation of fibrin clots [4]. Originally introduced in the 1960s, TXA has since demonstrated efficacy in reducing perioperative blood loss and transfusion requirements across various surgical disciplines, including orthopedic, cardiac, trauma, and obstetric surgeries [5-7]. The CRASH-2 and WOMAN trials highlighted the role of TXA in reducing mortality due to hemorrhage when administered early in trauma and postpartum hemorrhage, respectively [8-9].

Despite its widespread application, the evidence base for TXA in burn surgery remains limited and fragmented. Burn-related coagulopathy, vascular injury, and the hypermetabolic state following major burns contribute to complex hemostatic alterations, which may modify the pharmacodynamics and efficacy of antifibrinolytic agents like TXA in this patient population [10]. Moreover, burn surgeries differ substantially from other procedures due to their prolonged nature, extensive tissue manipulation, and high risk of blood loss. These unique considerations underscore the need for focused evaluation of TXA in the context of burn care.

While some randomized controlled trials and observational studies have explored the use of TXA in burn surgery, findings have been inconsistent. Some studies report a reduction in intraoperative blood loss and transfusion requirements, while others fail to demonstrate significant benefits or raise concerns regarding thromboembolic risks [11-12]. The heterogeneity in study designs, dosing regimens, surgical techniques, and outcome measures further complicates the interpretation of available evidence.

Given this uncertainty, a systematic review and meta-analysis are warranted to synthesize the current literature and provide a comprehensive assessment of efficacy and safety of TXA in burn patients undergoing surgical intervention. This study aims to evaluate the impact of TXA on intraoperative blood loss, transfusion needs, and adverse outcomes in this unique patient population. The findings are expected to inform clinical practice and guide future research on hemostatic strategies in burn surgery.

## Review

Methodology 

Information Sources and Search Strategy 

We conducted a comprehensive literature search across four electronic databases: PubMed, Embase, Web of Science, and the Cochrane Central Register of Controlled Trials (CENTRAL) from inception to June 5, 2025. The search strategy combined Medical Subject Headings (MeSH) and free-text terms related to “tranexamic acid,” “burn,” “burn surgery,” and “blood loss.” The search was restricted to studies published in English. In addition, we manually screened the reference lists of included studies and relevant reviews to identify any additional eligible studies.

*Study Selection* 

Two independent reviewers screened the titles and abstracts of all retrieved articles. Studies were eligible for inclusion if they met the following criteria: (1) randomized controlled trials (RCTs), (2) involved burn patients undergoing surgical procedures such as excision and grafting, (3) compared tranexamic acid to placebo or no intervention, and (4) reported at least one of the following outcomes: total blood loss, change in hemoglobin, or change in hematocrit. Studies involving non-burn surgical populations or lacking relevant outcome data were excluded. Full-text articles were assessed independently by the same reviewers. Discrepancies were resolved by consensus or consultation with a third reviewer.

Data Extraction and Outcomes 

Data were extracted independently by two reviewers using a standardized data extraction form. Extracted information included study characteristics (first author, year, country, study design), patient demographics including age, sex, and body mass index (BMI), intervention details (TXA dosage), and outcomes of interest. The primary outcomes were total blood loss (measured intraoperatively), change in hemoglobin levels (pre- and post-operative), and change in hematocrit levels.

Quality Assessment 

The risk of bias for included randomized controlled trials was assessed using the Cochrane Risk of Bias 2.0 (RoB 2) tool. The domains evaluated included randomization process, deviations from intended interventions, missing outcome data, measurement of outcomes, and selection of reported results.

Statistical Analysis Plan 

Meta-analyses were performed using Review Manager (RevMan, Cochrane, London, United Kingdom) version 5.4. For continuous outcomes (total blood loss, hemoglobin change, and hematocrit change), we calculated the mean difference (MD) with 95% confidence intervals (CIs). A random-effects model was used to account for between-study heterogeneity. Statistical heterogeneity was assessed using the I² statistic, with values >50% indicating substantial heterogeneity. Where applicable, sensitivity analyses were conducted by excluding studies at high risk of bias. Publication bias was not assessed because the number of included studies was less than 10.

Results 

**Figure 1:**
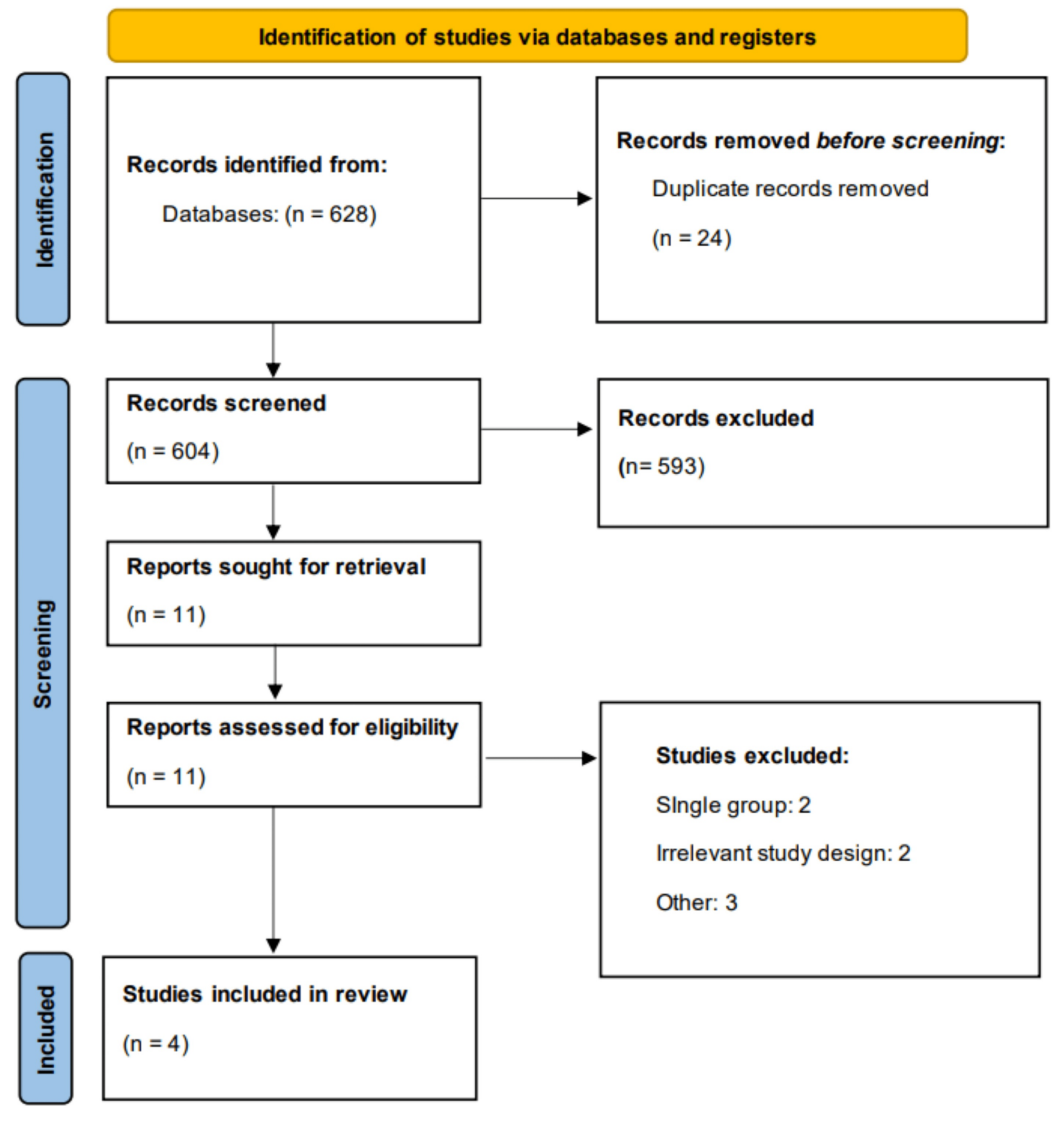
Study selection flowchart

**Figure 2:**
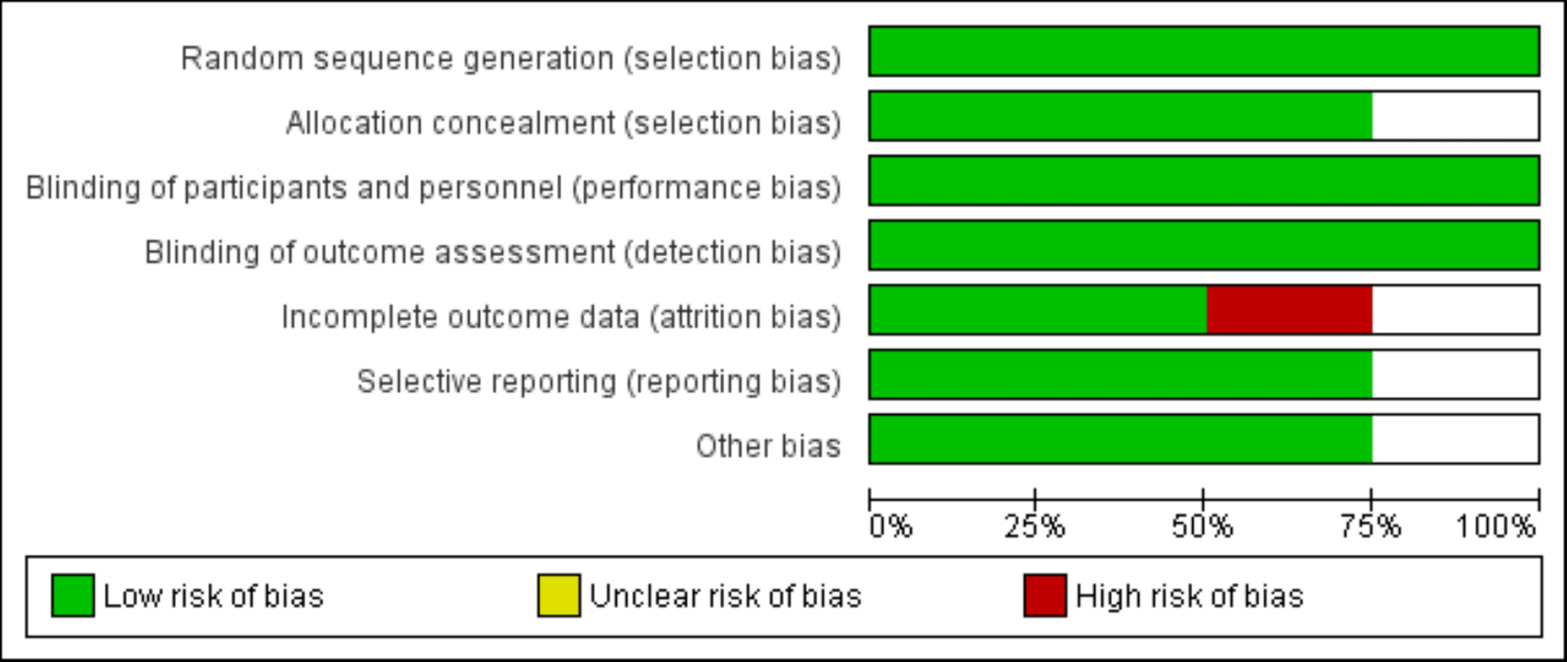
Risk of bias graph

A total of 628 records were retrieved from the initial database search. After removing duplicates and screening titles and abstracts, 11 full-text articles were assessed for eligibility. Of these, four studies met the inclusion criteria and were included in the final analysis. Detailed selection process is shown in Figure 1. Table 1 presents characteristics of included studies. The total sample size across studies was 264 patients, with 130 receiving TXA and 134 in the control groups. TXA was administered intravenously in doses ranging from 10 mg/kg to 15 mg/kg. Figure 2 presents quality assessment of included studies.

**Table 1:** Characteristics of included studies TXA: tranexamic acid.

Author	Year	Region	Groups	Dose of TXA	Sample Size	Mean Age	Male (n)	Mean BMI
Ajai et al. [13]	2022	India	TXA	15 mg/kg	15	33.6	12	21.2
			Control		15	30.7	14	20.9
Bhatia et al. [14]	2017	India	TXA	15 mg/kg	25	35.12	15	20.8
			Control		25	36.16	14	21.6
Castillo-Cardiel et al. [15]	2025	Mexico	TXA	10 mg/kg	15	33.8	9	27.3
			Control		15	35.2	11	26.2
Naderi et al. [16]	2025	Iran	TXA	10 mg/kg	47	39.3	24	24.8
			Control		47	40.1	25	24.5

*Loss of Blood During Surgery* 

**Figure 3:**
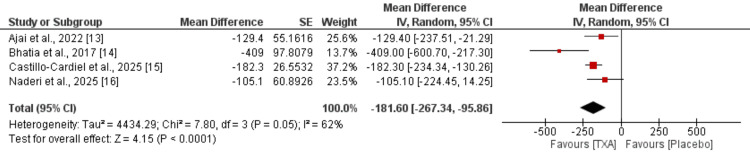
Comparison of blood loss during surgery Sources: References [13-16]. TXA: tranexamic acid.

Four randomized controlled trials reported data on intraoperative blood loss comparing tranexamic acid (TXA) to placebo. The pooled analysis showed that TXA significantly reduced blood loss, with a mean difference of -181.60 mL (95% CI: -267.34 to -95.86; p < 0.0001) as shown in Figure 3. There was moderate heterogeneity among studies (I² = 62%, p = 0.05). The largest effect was observed in the study by Bhatia et al. [14], while all studies demonstrated a reduction in blood loss favoring TXA. Overall, the findings suggest a substantial hemostatic benefit of TXA in burn patients undergoing surgical procedures.

Effect of Tranexamic Acid on Hemoglobin Levels 

**Figure 4:**
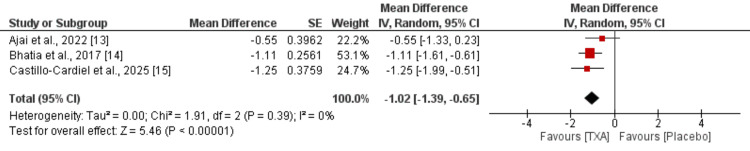
Comparison of hemoglobin Sources: References [13-15]. TXA: tranexamic acid.

Three randomized controlled trials evaluated the impact of tranexamic acid (TXA) on perioperative hemoglobin reduction. The pooled analysis demonstrated that TXA was associated with a significantly smaller decline in hemoglobin levels compared to placebo, with a mean difference of -1.02 g/dL (95% CI: -1.39 to -0.65; p < 0.00001) as shown in Figure 4. The analysis showed no heterogeneity across the studies (I² = 0%, p = 0.39), indicating consistent findings. These results reinforce the efficacy of TXA in minimizing hemoglobin loss during surgical interventions in burn patients.

Effect of Tranexamic Acid on Hematocrit Levels

**Figure 5:**
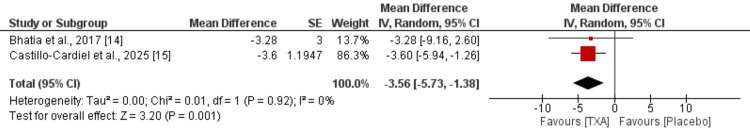
Comparison of hematocrit Sources: References [14-15]. TXA: tranexamic acid.

Three randomized controlled trials evaluated the impact of TXA on perioperative hematocrit reduction. The pooled analysis demonstrated that TXA was associated with a significantly smaller decline in hematocrit levels compared to placebo, with a mean difference of -1.02 g/dL (95% CI: -1.39 to -0.65; p < 0.00001) as shown in Figure 5. The analysis showed no heterogeneity across the studies (I² = 0%, p = 0.39), indicating consistent findings.

Discussion 

This meta-analysis aimed to evaluate the hemostatic efficacy of tranexamic acid (TXA) in patients with burn injuries undergoing surgical procedures. We included four randomized controlled trials (RCTs), encompassing a total of 264 participants. Our findings indicate that the use of TXA was associated with a significant reduction in intraoperative blood loss. Furthermore, patients in the TXA group exhibited smaller declines in hemoglobin and hematocrit levels compared to those who received placebo or no intervention. These results suggest that TXA may confer meaningful clinical benefits in minimizing perioperative bleeding in burn surgery.

Contrary to our findings, a prior meta-analysis conducted by Slob et al., (2024) [12] did not demonstrate a statistically significant reduction in blood loss with TXA administration. However, that review was limited by the inclusion of only two RCTs, whereas our analysis incorporated four trials, thereby offering a more comprehensive and updated synthesis of evidence. Another meta-analysis by Fijany et al. [17] reported a significant reduction in perioperative blood loss with TXA using a random-effects model. Nevertheless, their analysis included both RCTs and non-randomized studies, potentially introducing confounding due to differences in study design. In contrast, our decision to exclusively include RCTs enhances internal validity, as randomization helps balance known and unknown confounders across intervention groups.

The role of TXA in reducing blood loss and transfusion requirements has been well-documented across numerous surgical specialties, although burn surgery presents unique challenges with its characteristically prolonged operative times and extensive tissue disruption that may influence TXA's effectiveness and dosing requirements. A 2021 meta-analysis that pooled data from 57 RCTs across orthopedic, obstetric, cardiac, maxillofacial, and plastic surgeries demonstrated consistent efficacy of TXA in minimizing surgical bleeding [18]. Additionally, TXA has been shown to reduce bleeding-related mortality, particularly in trauma and postpartum hemorrhage settings, with a favorable safety profile [19,20]. Evidence suggests that TXA can reduce surgical blood loss by nearly one-third compared to placebo [20].

In the context of burn surgery, TXA may also interact with the unique pathophysiological processes involved in wound healing. The systemic inflammatory response following burn injury is essential for initiating tissue repair. However, TXA’s anti-inflammatory properties may theoretically dampen this response, potentially affecting wound healing [21]. While this concern remains speculative, it underscores the importance of studying the broader physiological impact of TXA in burn patients.

Safety outcomes related to TXA use have gained increasing attention. A large-scale study evaluating thromboembolic risk in patients with traumatic brain injury found no significant difference in venous thromboembolism between TXA and control groups [22]. In our included studies, no serious adverse events related to TXA were reported. However, the small sample sizes and limited follow-up in these trials restrict their ability to detect rare or delayed complications.

Another limitation of our meta-analysis is the lack of data on the timing of TXA administration relative to the burn injury. Previous research has emphasized that timing can influence both the safety and efficacy of TXA. The CRASH-2 trial, for example, demonstrated optimal outcomes when TXA was administered within three hours of trauma [23]. In the context of burn surgery, where operations may occur days or weeks post-injury, the temporal relevance of TXA administration remains unclear. Therefore, the generalizability of our findings is constrained by the absence of data on this critical variable.

Although current evidence supports the hemostatic benefit of TXA in burn surgery, robust multicenter RCTs with larger sample sizes are needed to validate these findings and assess other clinically important endpoints such as transfusion requirements, graft viability, wound healing, and patient-centered outcomes. Future research should also explore the differential effects of topical versus systemic TXA administration, examine the long-term impact on graft survival, assess gender-specific responses, and clarify the influence of timing between injury and TXA administration on surgical outcomes.

Study Limitations

This meta-analysis has several limitations. First, the number of included studies and overall sample size were relatively small, limiting the statistical power and generalizability of the findings. Second, heterogeneity in TXA dosage, timing of administration, and surgical techniques may have influenced the outcomes. Third, none of the included studies were powered to detect rare adverse events, limiting our ability to assess the safety profile of TXA. Lastly, important variables such as the time interval between burn injury and surgery were not consistently reported, which may affect the interpretation of TXA’s effectiveness in different clinical settings.

## Conclusions

This systematic review and meta-analysis provides compelling evidence supporting the hemostatic efficacy of tranexamic acid in burn surgery. Our findings demonstrate that TXA administration significantly reduces intraoperative blood loss and minimizes perioperative hemoglobin decline compared to placebo or no intervention. These results suggest that TXA offers meaningful clinical benefits in managing surgical bleeding during burn procedures, potentially reducing transfusion requirements and associated complications. The consistency of findings across included studies, particularly for hemoglobin outcomes with zero heterogeneity, strengthens confidence in these results. However, several limitations warrant consideration, including the relatively small sample size, heterogeneity in dosing regimens, and insufficient data on the timing of administration relative to burn injury. Future research should focus on large-scale multicenter trials to validate these findings, establish optimal dosing protocols, evaluate long-term safety outcomes, and assess the impact on clinically relevant endpoints such as transfusion requirements and patient-centered outcomes in burn surgery.
